# Analysis of clinical application of thoracoscopic lobectomy for lung cancer

**DOI:** 10.1186/1477-7819-12-157

**Published:** 2014-05-21

**Authors:** Qing-Quan Luo, Hao Lin, Qiang Tan, Jia Huang, Lin Xu

**Affiliations:** 1 Shanghai Lung Cancer Clinical Medical Center, Shanghai Chest Hospital, Shanghai Jiaotong University, 200030 Shanghai, China; 2 Department of Thoracic Surgery, Nanjing Medical University Affiliated Cancer Hospital, No.42 Bai zi ting Road, Jiangsu 210009 Nanjing, China; 3 Nanjing Medical University, Nanjing 210008, Jiangsu Province, China

**Keywords:** Video-assisted thoracoscopic surgery (VATS), Clinical early stage lung cancer, Lobectomy, Complication, Short-term recurrence rate

## Abstract

**Background:**

VATS has been extensively considered as a standard method of pulmonary diagnosis and treatment of benign lung diseases. This study aimed to investigate the safety, efficacy, and feasibility of video-assisted thoracoscopic surgery (VATS) lobectomy compared with conventional lobectomy via open thoracotomy in patients with clinical early stage lung cancer.

**Methods:**

A total of 120 patients with lung cancer underwent VATS lobectomy; another 120 patients with lung cancer underwent conventional lobectomy. The clinical outcomes from these two groups were retrospectively analyzed and compared.

**Results:**

The numbers of patients who underwent lobectomy in the left upper lobe, left lower lobe, right upper lobe, right middle lobe, and right lower lobe were 24, 28, 40, 4, and 24 in the VATS group and 38, 20, 30, 7, and 25 in the conventional group, respectively. No statistical differences were observed between the two groups. Likewise, no statistical differences were observed in terms of duration of operation, time for postoperative extubation, complications, length of hospital stay, and number of dissected lymph nodes (VATS group: left, 5.12 ± 1.45, right, 6.84 ± 1.33; conventional group: left, 4.96 ± 1.39 mm, right, 6.91 ± 1.27; *P* >0.05).

**Conclusion:**

Anatomical lobectomy was successfully completed by VATS lobectomy for lung cancer; the standard lymph node dissection was also achieved. This procedure also showed advantages in terms of surgical bleeding, duration, postoperative complications, indwelling time of chest tube, and short-term recurrence rate without significant differences from conventional lobectomy.

## Background

Video-assisted thoracoscopic surgery (VATS) was first applied in the diagnosis and treatment of lung diseases in the early 1990s. VATS has been gradually accepted in other applications, such as local resection of pulmonary nodules and treatment of early stage lung cancer, among others [1-4]. VATS has been extensively considered as a standard method of pulmonary diagnosis and treatment of benign lung diseases [5,6].

VATS lobectomy has been performed by thoracic surgeons to treat clinical early stage non-small cell lung cancer (NSCLC) because this method shows several advantages, such as shorter postoperative hospital stay, slight pain, less intraoperative and postoperative complications, and less loss of lung function in patients compared with conventional lobectomy [6,7].

We conducted a retrospective study of patients with clinical early stage lung cancer and underwent VATS lobectomy in our department. We then analyzed lymph node dissection, recurrence rate, survival rate, and intraoperative and postoperative complications. We also investigated the safety, efficacy, and feasibility of VATS lobectomy compared with conventional lobectomy via open thoracotomy in patients within the same period.

## Methods

### Clinical data

We retrospectively analyzed and compared the clinical outcomes of 120 patients with NSCLC and underwent VATS lobectomy as well as those of 120 patients with clinical early stage lung cancer and underwent conventional lobectomy between January 2007 and July 2009. The inclusion criteria were listed as follows: clinical early stage NSCLC; peripheral lesions with a diameter <3 cm in the CT image; no evident swollen lymph nodes in the mediastinum; and no distant metastasis. We also performed preoperative routine examinations, including chest computed tomography (CT), fiber bronchoscopy, brain CT/brain magnetic resonance imaging (MRI), whole-body bone scan, PET-CT, and abdominal ultrasound, among others, to evaluate lesions comprehensively and exclude distant metastases or organic diseases in vital organs, such as the heart and lungs. Cardiopulmonary function was checked to assess surgical risk. Anesthesia was applied similarly in VATS lobectomy and conventional lobectomy (double cavity bronchial intubation, ventilator-assisted one-lung ventilation, and balanced intravenous anesthesia). The basic information and imaging of the patients in the two groups are shown in Table 1. This study was conducted in accordance with the declaration of Helsinki. This study was conducted with approval from the Ethics Committee of Shanghai Chest Hospital, Shanghai Jiaotong University. Written informed consent was obtained from all participants.

**Table 1:** Basic data of patients

**Item**			**Groups**	
			VATS	Conventional
Cases (male/female)			120 (56/64)	120 (70/50)
Age (year)			59.35 ± 8.96	61.40 ± 10.33
Lesion location	LU		24	38
	LL		28	20
	RU		40	30
	RM		4	7
	RL		24	25
Characteristic of lesion	GGO		14	2
	Nodules		18	3
	Mass		88	115
Pathological type	ad		85	87
	scc		32	29
	ad-sq		2	3
	lc		1	1
Tumor size (maximal diameter, cm)			2.98 ± 1.76	3.11 ± 1.44
Pathological stage	Ia		20	12
	Ib		73	84
	IIb		11	10
	IIIa		16	14
Lymph nodes dissection	Left side	Range	5-11	5-11
		Average	5.12 ± 1.45	4.96 ± 1.39
	Right side	Range	2-4, 7-11	2-4, 7-11
		Average	6.84 ± 1.33	6.91 ± 1.27

### VATS lobectomy

VATS lobectomy was performed under thoracoscope monitoring with the aid of three to four holes, also known as small incisions, on the chest wall.

The main incision was made approximately 4 cm from the anterior axillary line to the posterior axillary line without a spreader. The main incision was between the fourth rib of anterior axillary line. The length of incision was based on the size of lung lesions and tumors; an incision 1 cm to 2 cm longer than the main incision was necessary to dissect larger lung lesions and tumors. Most of our procedures were completed through this incision, which was anatomically located opposite the oblique fissure of the lung and the superior pulmonary vein, with a short straight line distance from the hilus pulmonis, thereby enabling an ordinary instrument to reach the hilus pulmonis and complete the anatomical resection of both upper and lower lobes, as well as the superior mediastinal lymph nodes, with the aid of an auxiliary incision.

The auxiliary incision (the second incision) was 2 cm to 3 cm long at the fifth intercostal space inferior to the scapula (two fingers below the infrascapular line, which was about in the sixth intercostal space), opposite the posterior oblique fissure and carina region. This incision was made to expose the lung tissue, facilitate the separation of the sheath membrane of the interlobar vessels by using ordinary vessel forceps, and enable conversion from thoracoscopic surgery to conventional surgery. The same incision was also appropriate for vessel forceps and oval forceps; furthermore, this incision was used to dissect the lymph node in the carina region.

### Right upper lobectomy

Right upper lobectomy was performed starting from the right upper pulmonary vein; if the interlobar fissure was well developed, the sheath membrane of the interlobar arteries could be initially isolated until the anterior hilus pulmonis was reached; the interlobar fissure tissue was cut open using an automatic cutting stapler. The posterior ascending arteries were then processed. The lung was pulled toward the posterosuperior direction to expose the bronchus in the right upper lobe, and the interlobar lymph nodes were removed. The step order was determined based on the positions of the bronchus and the truncus anterior. The surgical procedures were similar to those for thoracotomy. Right upper lobectomy could be performed in the following order: vein, artery, bronchus, and interlobar fissure.

Left upper lobectomy was performed initially from the left upper pulmonary vein. If the interlobar fissure was well developed, the sheath membrane of the interlobar vessels could be isolated until the interstitium of the pulmonary hilum was reached. The interlobar fissure tissue was then cut open using an automatic cutting stapler. Ascending and lingual segmental arteries were exposed using a cutting stapler, thereby clearing the upper lobe bronchus and facilitating easy operation. The truncus anterior was finally dissected.

Right lower lobectomy was performed by initially dividing the inferior pulmonary ligament and dissecting the lymph nodes of groups 8 and 9 to expose the inferior pulmonary vein, which was then enveloped with a rubber loop. If the interlobar fissure was well developed, the sheath membrane of the interlobar arteries could be isolated until the anterior hilus pulmonis and the interlobar fissure tissue were completely separated from the lobi medius pulmonis by using an automatic cutting stapler. This procedure should be performed with caution to prevent injury to the right middle lobe pulmonary vein. The relation between the right lower lobe pulmonary arteries (separated or combined dissection of dorsal arteries and basal segmental arteries could be performed) and the bronchus was clearly shown, particularly the position of the middle lobe bronchus, which facilitated the surgical procedure on the right lower lobe bronchus. The right lower lobe pulmonary arteries and the interlobar fissure tissue were finally dissected.

Left lower lobectomy first exposed the inferior pulmonary vein that was then enveloped with a rubber loop. If the interlobar fissure was well developed, the sheath membrane of the interlobar arteries could be isolated until the anterior hilus pulmonis and interlobar fissure tissue were completely separated from the lingual segment of the left upper pulmonary tissue. This procedure should also be performed with caution to avoid injury to the right upper lobe pulmonary vein. The relation between the right lower lobe pulmonary arteries (dorsal arteries and basal segmental arteries could be dissected separately or together) and the bronchus was clearly shown, facilitating the surgical procedure on the left lower lobe bronchus. The left lower lobe pulmonary arteries and the interlobar fissure tissue were finally dissected.

### Research contents

We compared different parameters, including surgical bleeding, lymph node dissection, duration of surgery, indwelling time of chest tube, postoperative complications and hospital stay, short-term recurrence rate, and survival rate to investigate the safety, efficacy, and feasibility of VATS lobectomy in patients with lung cancer.

### Statistical analysis

The distribution map of the metastatic lymph nodes [7] was employed in both VATS and conventional groups (N1, groups 10 to 14; N2, groups 1 to 9). The pathological stage was classified according to the 1997 TNM system. The frequency distribution was analyzed using a chi-square test. Data between groups were compared using a two-tailed *t*-test. Survival curves were compared by the use of Kaplan-Meier analysis and Loglank test. *P* <0.05 was considered statistically significant.

## Results and discussion

### Results

#### General data of surgery

Operation time, operative bleeding volume, length of postoperative drainage, and postoperative hospitalization in the two groups are shown in Table 2. No intraoperative or perioperative death occurred in both groups.

**Table 2:** Information related with operation

**Groups**	**Operation time (min)**	**Postoperative drainage (day)**	**Operative bleeding volume (mL)**	**Postoperative hospitalization (day)**
VATS	135 ± 24.4	4.4 ± 4.16	121 ± 94.0	7.9 ± 5.1
Conventional	130 ± 27.0	4.5 ± 3.97	253 ± 89.1	9.8 ± 6.0
*P* value	>0.05	>0.05	<0.001	>0.05

#### Follow-up

The postoperative follow-up ranged from 6 months to 36 months, with follow-up rates of 94.2% in the VATS group and 91.7% in the conventional group. Three cases of recurrence (2.5%) occurred in the VATS group; these cases were similar to the four cases (3.3%) in the conventional group. However, two cases (1.67%) of distal metastasis (liver, adrenal gland) were observed in the VATS group; this result was significantly different from that in the six cases (two cases of liver, two cases of bone, one case of brain, and one case of adrenal gland; 5%) in the conventional group. The survival rates of patients in the VATS and conventional groups were 99% *versus* 98%, respectively, for 1 year; these rates were, respectively, 93.9% *versus* 92.7% for 3 years (Table 3).

**Table 3:** Survival rate

**Groups**	**Local recurrence**	**Systematic metastasis**	**1-year survival**	**3-year survival**
VATS	3	2	99.0%.	93.9%
Conventional	4	6	98.0%	92.7%
*P* value	>0.05	0.013	>0.05	>0.05

#### Complications

Intraoperative complications included conversion to an open thoracotomy during VATS lobectomy in four cases (3.33%), among which two cases (1.67%) were attributed to pleural adhesion; two other cases (1.67%) were caused by uncontrolled bleeding.

Postoperative complications included atrial fibrillation in 21 cases (17.5%) in the VATS group and 24 cases (20.0%) in the conventional group (*P* >0.05). The comparison of postoperative complications is shown in Table 4.

**Table 4:** Complication

**Groups**	**Intensive pain**	**Atrial fibrillation**	**Prolonged air leak**	**BPF**	**Others**	**Total**
VATS	2	21	2	1	0	26
Conventional	1	24	2	1	1	29

No significant difference between two groups (*P* >0.05).

Persistent air leak was observed in three cases (2.5%) in the VATS group and two cases (1.67%) in the conventional group. This leak was resolved at 10 and 12 days in VATS and conventional groups by continuous negative pressure suction with a water-sealed bottle; the chest tube was then pulled out.

One case (0.83%) of bronchopleural fistula (BPF) was observed in each group. A male patient with stage IIb NSCLC in the VATS group and a male patient with stage Ib NSCLC in the conventional group underwent right lower lobectomy.

### Discussion

VATS lobectomy or thoracoscopic lobectomy without rib spreading, in the domain of minimally invasive surgery, has been performed worldwide since this procedure was introduced to chest surgery in the 1990s.

Several reasons caused the conversion to thoracotomy during VATS lobectomy; these reasons include bleeding, pleural adhesion, bronchial injury, and contralateral pneumothorax [8,9]. The intraoperative conversion rate was 0% to 15% (median, 8.1%). In our study, conversion to an open thoracotomy during VATS lobectomy occurred in four cases (3.33%), in which two cases were attributed to pleural adhesion and another two were because of uncontrolled bleeding. Timely treatment avoided irreversible consequences.

Pleural adhesions have been considered by authors as the most common reason for conversion to thoracotomy during VATS lobectomy in studies on thoracoscopy surgery. We discovered 62 cases (51.7%) of pleural adhesions in the VATS group, including 46 cases (38.3%) of mild adhesions (on parietal, mediastinal, and diaphragmatic surfaces), 14 cases (11.7%) of moderate adhesions (more than two sites, but lysis of pleural adhesions could be performed with a thoracoscope and instruments), and two cases (1.67%) of severe adhesions (conversion to thoracotomy). Therefore, the patients with conversion to thoracotomy accounted for 3.23% (2/62) of all patients with pleural adhesion complications. This result indicated that lysis of pleural adhesions was effective in many cases during VATS lobectomy.

In errhysis or postoperative drainage, no patient underwent a second thoracotomy because of a large amount of postoperative drainage in our study. Other studies have suggested different indwelling days of chest tube after VATS lobectomy; two of these studies have reported a significantly shorter time, whereas others have not shown a distinct difference from our study [10].

Although VATS lobectomy is a minimally invasive surgery, postoperative pain is inevitable as in other operations. In the VATS group, one patient (0.83%) needed an intercostal nerve block during hospital stay, and another patient (0.83%) underwent treatment for postoperative pain after 1 month follow-up period; this procedure was consistent with that in the conventional group. Studies have revealed that postoperative intercostal neuralgia or intercostal neuritis may result from cannula insertion and friction between incisions and a thoracoscope [10,11].

To determine atrial fibrillation, we performed electrocardiogram (ECG) monitoring before the chest tube was pulled out. Atrial fibrillation was observed in 21 cases (17.5%, including one patient with a 3-year history of atrial fibrillation but without daily symptoms) in the VATS group and 24 cases (20.0%) in the conventional group (*P* >0.05). Clinical symptoms were not observed in any patient, but atrial fibrillation waves were shown by ECG. No statistical difference in the incidence of atrial fibrillation was found between VATS and conventional groups; this result is consistent with that in previous studies [12-15].

Persistent air leak was discovered in two cases (1.67%). The cases were cured at 10 and 12 days after surgery by continuous negative pressure suction with a water-sealed bottle; afterward, the chest tube was pulled out. The patients were discharged from the hospital after a review of posterior-anterior chest X-ray film without discomfort. Two patients (1.67%) with persistent air leak in the conventional group were discharged from the hospital after the same treatment. Air probably leaked from the endpoint of incision, despite being reinforced, during operating on an incomplete oblique fissure with endoscopic devices. Studies have also revealed a persistent air leak incidence of 1% [14], which was similar to our result.

One case (0.83%) of BPF was found in each group. The patients had a smoking history (40 cigarettes/1,200 cigarettes per year and 30 cigarettes/750 cigarettes per year) and underwent a right lower lobectomy.

No statistical differences in local recurrence rate, 1-year survival rate, and 3-year survival rate were observed, but the distal metastasis rate in the VATS group was superior to that of the conventional group; this result is consistent with that in previous studies [2,16-19]. The International Society for Minimally Invasive Cardiothoracic Surgery published a consensus statement on thoracoscopic lobectomy in 2007 [20], recommending thoracoscopic lobectomy for patients with clinical stages I and II NSCLC.

## Conclusion

Our result showed the satisfactory efficacy and safety of VATS lobectomy for clinical early stage NSCLC (peripheral lesions). VATS lobectomy satisfied the criteria for surgical treatment of lung cancer. This procedure can possibly become a standard mode of surgery for peripheral NSCLC.

## Competing interests

The authors declare that they have no competing interests.

## Authors’ contributions

QL and HL participated in the design of the study, performed the statistical analysis, and drafted the manuscript. QT and JH participated in study design and literature search. LX conceived of the study, participated in the design, and helped to draft the manuscript. All authors read and approved the final manuscript.
